# Supplementary material for: Modeling glioblastoma heterogeneity as a dynamic network of cell states
Source: Mol Syst Biol. 2021 Sep 16;17(9):e10105. doi: 10.15252/msb.202010105 (PMC8444284; doi:10.15252/msb.202010105)
Supplement: Supplementary file 6 — Source Data for Figure 5 [file MSB-17-e10105-s004.zip › Figure5A_sourcedata/GSEA_3071/hallmarks_stateIII.GseaPreranked.1623742540963/HALLMARK_GLYCOLYSIS.html]

Details for gene set HALLMARK\_GLYCOLYSIS[GSEA]

|  || Dataset | state33071 |
| Phenotype | NoPhenotypeAvailable |
| Upregulated in class | na\_neg |
| GeneSet | HALLMARK\_GLYCOLYSIS |
| Enrichment Score (ES) | -0.2835277 |
| Normalized Enrichment Score (NES) | -1.5735402 |
| Nominal p-value | 0.033732317 |
| FDR q-value | 0.09903698 |
| FWER p-Value | 0.808 |
Table: GSEA Results Summary

  

Fig 1: Enrichment plot: HALLMARK\_GLYCOLYSIS      
 Profile of the Running ES Score & Positions of GeneSet Members on the Rank Ordered List

  

| PROBE | GENE SYMBOL | GENE\_TITLE | RANK IN GENE LIST | RANK METRIC SCORE | RUNNING ES | CORE ENRICHMENT || 1 | TXN |  |  | 47 | 0.465 | 0.0071 | No |
| 2 | MIF |  |  | 68 | 0.445 | 0.0203 | No |
| 3 | PPIA |  |  | 121 | 0.395 | 0.0232 | No |
| 4 | ENO1 |  |  | 241 | 0.314 | 0.0055 | No |
| 5 | STMN1 |  |  | 298 | 0.291 | 0.0030 | No |
| 6 | SOD1 |  |  | 315 | 0.287 | 0.0108 | No |
| 7 | MDH2 |  |  | 393 | 0.262 | 0.0017 | No |
| 8 | HK2 |  |  | 432 | -0.250 | 0.0023 | No |
| 9 | ALDOA |  |  | 440 | -0.251 | 0.0108 | No |
| 10 | PLOD2 |  |  | 521 | -0.255 | 0.0007 | No |
| 11 | GPC4 |  |  | 593 | -0.259 | -0.0069 | No |
| 12 | GUSB |  |  | 641 | -0.262 | -0.0082 | No |
| 13 | PC |  |  | 1064 | -0.283 | -0.1055 | No |
| 14 | XYLT2 |  |  | 1133 | -0.286 | -0.1112 | No |
| 15 | GALK2 |  |  | 1139 | -0.287 | -0.1007 | No |
| 16 | DEPDC1 |  |  | 1564 | -0.310 | -0.1973 | No |
| 17 | PHKA2 |  |  | 1584 | -0.311 | -0.1894 | No |
| 18 | DPYSL4 |  |  | 1761 | -0.322 | -0.2215 | No |
| 19 | NT5E |  |  | 1769 | -0.323 | -0.2100 | No |
| 20 | GYS1 |  |  | 1894 | -0.329 | -0.2284 | No |
| 21 | ZNF292 |  |  | 1992 | -0.335 | -0.2396 | No |
| 22 | CAPN5 |  |  | 1994 | -0.335 | -0.2260 | No |
| 23 | DDIT4 |  |  | 2027 | -0.337 | -0.2203 | No |
| 24 | PLOD1 |  |  | 2056 | -0.338 | -0.2136 | No |
| 25 | NANP |  |  | 2076 | -0.340 | -0.2044 | No |
| 26 | MET |  |  | 2138 | -0.344 | -0.2060 | No |
| 27 | MXI1 |  |  | 2182 | -0.347 | -0.2027 | No |
| 28 | SLC37A4 |  |  | 2200 | -0.349 | -0.1927 | No |
| 29 | GMPPB |  |  | 2230 | -0.351 | -0.1857 | No |
| 30 | ENO2 |  |  | 2366 | -0.363 | -0.2055 | No |
| 31 | CD44 |  |  | 2471 | -0.371 | -0.2170 | No |
| 32 | SDC2 |  |  | 2537 | -0.376 | -0.2183 | No |
| 33 | KIF20A |  |  | 2561 | -0.379 | -0.2086 | No |
| 34 | STC2 |  |  | 2570 | -0.379 | -0.1949 | No |
| 35 | QSOX1 |  |  | 2574 | -0.380 | -0.1800 | No |
| 36 | HMMR |  |  | 2940 | -0.413 | -0.2572 | No |
| 37 | PAM |  |  | 3043 | -0.425 | -0.2660 | Yes |
| 38 | CHPF |  |  | 3085 | -0.429 | -0.2588 | Yes |
| 39 | P4HA2 |  |  | 3174 | -0.441 | -0.2633 | Yes |
| 40 | HSPA5 |  |  | 3193 | -0.442 | -0.2497 | Yes |
| 41 | ALG1 |  |  | 3291 | -0.458 | -0.2558 | Yes |
| 42 | ANKZF1 |  |  | 3302 | -0.460 | -0.2394 | Yes |
| 43 | CTH |  |  | 3386 | -0.478 | -0.2411 | Yes |
| 44 | TFF3 |  |  | 3413 | -0.483 | -0.2278 | Yes |
| 45 | EGFR |  |  | 3433 | -0.488 | -0.2125 | Yes |
| 46 | ABCB6 |  |  | 3522 | -0.516 | -0.2139 | Yes |
| 47 | NDST3 |  |  | 3548 | -0.526 | -0.1987 | Yes |
| 48 | SOX9 |  |  | 3560 | -0.531 | -0.1795 | Yes |
| 49 | TPBG |  |  | 3638 | -0.570 | -0.1759 | Yes |
| 50 | B3GAT1 |  |  | 3683 | -0.601 | -0.1624 | Yes |
| 51 | DCN |  |  | 3723 | -0.633 | -0.1463 | Yes |
| 52 | VCAN |  |  | 3741 | -0.660 | -0.1234 | Yes |
| 53 | STC1 |  |  | 3770 | -0.702 | -0.1016 | Yes |
| 54 | TGFBI |  |  | 3781 | -0.713 | -0.0747 | Yes |
| 55 | CXCR4 |  |  | 3802 | -0.750 | -0.0488 | Yes |
| 56 | COL5A1 |  |  | 3815 | -0.764 | -0.0204 | Yes |
| 57 | IGFBP3 |  |  | 3903 | -1.199 | 0.0067 | Yes |
Table: GSEA details [plain text format]

  

Fig 2: HALLMARK\_GLYCOLYSIS: Random ES distribution      
 Gene set null distribution of ES for **HALLMARK\_GLYCOLYSIS**
